# Validation of the PyloPlus Urea Breath Test System in pediatric patients

**DOI:** 10.1128/spectrum.03182-23

**Published:** 2023-12-05

**Authors:** Travis K. Price, Susan Realegeno, Kamran Azad, Kileen L. Shier

**Affiliations:** 1 Quest Diagnostics, Chantilly, Virginia, USA; 2 Quest Diagnostics, San Juan Capistrano, California, USA; University of Maryland School of Medicine, Baltimore, Maryland, USA

**Keywords:** *Helicobacter pylori*, urea breath test, pediatric, non-invasive

## Abstract

This study evaluated the performance of the PyloPlus Urea Breath Test (UBT) System, a Food and Drug Administration (FDA)-approved assay for diagnosis of *Helicobacter pylori* infection in adult patients, using samples from 111 pediatric patients (3–17 years old). Both qualitative results (100% correlation) and quantitative delta over baseline values (*R* ^2^ = 0.9989) from the PyloPlus UBT strongly correlated with the Meridian BreathTek POCone UBT reference method, which is FDA approved for use in pediatric patients (3–17 years old). The PyloPlus UBT performed comparably to the BreathTek UBT without any calculation correction factors for pediatric samples. Consistent with previous studies, male pediatric patients had a significantly higher positivity rate (64%) than female pediatric patients (33%) (*P* = 0.001). There was no difference in positivity rate between two performing locations. This study shows that the PyloPlus UBT system can be used for testing pediatric patient specimens.

**IMPORTANCE:**

For the diagnosis and post-treatment monitoring of *H. pylori* infection, non-invasive testing methodologies improve patient comfort, particularly for children. Previously, only the BreathTek UBT had FDA approval for use in pediatric patients and required an adjustment calculation based on age, height, and weight of the patient. The purpose of this study was to evaluate the performance of the PyloPlus UBT assay in a pediatric population.

## OBSERVATION

*Helicobacter pylori* is a Gram-negative bacterium that infects humans, contributing to peptic ulcer disease and gastric cancer if infection persists (1). The global prevalence in pediatric patients was estimated to be 32% as of 2021 (2). Clinical manifestations may differ in children compared to adults, and non-invasive testing is performed to evaluate patients presenting with specific clinical conditions or post-treatment eradication (3 – 5). Non-invasive testing for *H. pylori* infection utilizing non-radioactive labeled urea has improved post-therapy testing for *H. pylori* by avoiding invasive biopsy and culture. North American and European guidelines recommend two methods for non-invasive testing, which include a two-step monoclonal stool antigen test that is not currently available in the United States, and a urea breath test (UBT) (3, 4). Briefly, the UBT assay begins with the patient ingesting a ^13^C-urea supplement powder dissolved in water. The carbon-labeled urea is decomposed into ^13^CO_2_ and NH_3_ in the presence of urease associated with gastric *H. pylori* bacteria. ^13^CO_2_ is then absorbed into the blood and exhaled in the breath. Infrared spectrophotometry is used to compare the ratio of ^13^CO_2_ to ^12^CO_2_ in the collected breath specimens before and after ingestion of the ^13^C-urea supplement, as an indirect measure of *H. pylori* infection (6). The guidelines for the management of *H. pylori* in children and adolescents list the UBT as an acceptable non-invasive test for use in investigating causes of chronic immune thrombocytopenic purpura and post-treatment assessment in pediatric patients (3).

The PyloPlus Urea Breath Test System (Gulf Coast Scientific, Oldsmar, FL) is FDA approved for adult patients (≥18 years old) but not yet FDA approved for pediatric patients. In this study, the performance of the PyloPlus UBT assay in a pediatric patient population (3–17 years old) was evaluated.

## MATERIALS AND METHODS

Human breath specimens were collected from pediatric patient specimens (3–17 years old) submitted for routine clinical testing. BreathTek UBT Kit bags (Meridian Bioscience Corporation, Cincinnati, OH), one baseline and one post-dose sample bag, were tested using the Otsuka POCone infrared spectrophotometer system (Otsuka Pharmaceutical, Tokyo, Japan) at two performing sites (Quest San Juan Capistrano, CA, and Quest Chantilly, VA) according to the manufacturer’s instructions. The BreathTek UBT is FDA approved for pediatric patients (3–17 years old). Delta over baseline (DOB) results were calculated by measuring the difference between the ratio of ^13^CO_2_ to ^12^CO_2_ in the post-dose specimen and the corresponding baseline specimen. A subsequent urea hydrolysis rate (UHR) calculation [UHR (μg/min) = DOB × CO_2_ production rate × 0.3427], using a web-based pediatric urea hydrolysis rate calculation application, was performed. The UHR was used to adjust the DOB value based on relevant patient demographics including age, gender, height, and body weight. UHR values greater than or equal to 10 µg/min were reported as positive by the software.

After testing samples with the POCone system, remnant breath samples from each patient were transferred to an empty PyloPlus UBT bag (baseline or post-dose) using an adapter (5/16″ polyethene tubing) and tubing apparatus (3/8″ clear vinyl tubing). BreathTek bags were forcibly depressed to ensure air was completely transferred to the PyloPlus bag. PyloPlus bags were then tested by infrared spectrophotometry using the PyloPlus UBT Analyzer. DOB results were generated by the instrument based on the ^13^CO_2_ to ^12^CO_2_ ratio difference in the baseline versus post-dose bag. In accordance with the manufacturer’s instructions for adult patients, no additional calculations were performed, and DOB results greater than or equal to 3.0 were reported as positive.

Qualitative results generated by the BreathTek UBT and the PyloPlus UBT systems were compared. In addition, DOB values between the two systems were compared and linear regression was applied. Precision testing was performed using positive and negative samples tested in triplicate in 1 day (intra-assay) and in triplicate over 3 days (inter-assay). Separate samples were used between intra- and inter-assay testing due to limited availability of remnant specimen with sufficient volume.

## RESULTS

Samples from a total of 111 pediatric patients were analyzed with a mean age of 12.7 (SD = 3.7) and consisted of 66 (59%) female and 45 (41%) male patients. Fifty-one (46%) patients tested positive using the reference method (i.e.*,* BreathTek UBT System); 60 (54%) were negative. Sample positivity showed no correlation with age (*P* = 0.75) but did show a correlation with sex (*P* = 0.001) (Table 1).

**TABLE 1:** Summary of PyloPlus UBT results

	All (*N* = 111)	Positive (*N* = 51)	Negative (*N* = 60)	*P*-value^a^
Age	12.7 ± 3.7	12.8 ± 3.4	12.6 ± 3.9	0.75
Sex Male Female	45 66	29 (64%) 22 (33%)	16 (36%) 44 (67%)	0.001
Performing site San Juan Capistrano, CA Chantilly, VA	52 59	22 (42%) 29 (49%)	30 (58%) 30 (51%)	0.47

^a^ *P*-values represent comparison between positive and negative specimens. Age is presented as mean ± standard deviation. A two-tailed *t*-test was used to assess age. A χ^2^ test was used to assess sex and location.

**Fig 1:**
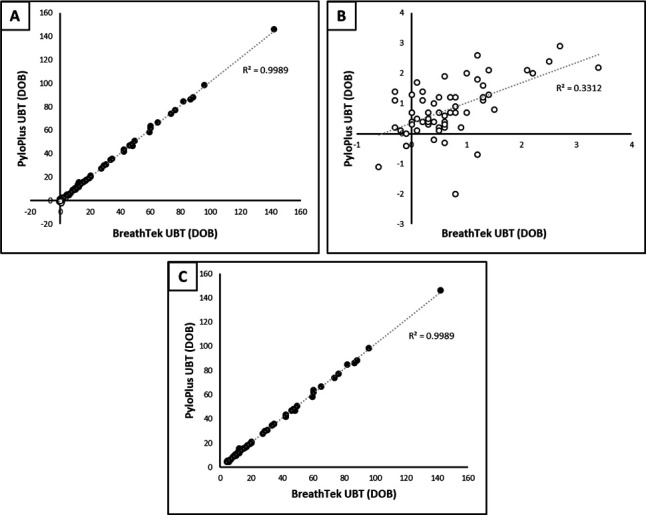
Comparison of DOB values between UBT assays. Positive (filled circles) and negative (open circles) DOB values are compared between the PyloPlus UBT and BreathTek UBT systems. Linear regression analysis represented by gray dotted line. (**A**) Comparison of all specimens (*N* = 111). (**B**) Comparison of negative specimens only (*N* = 60). (**C**) Comparison of positive specimens only (*N* = 51).

Qualitative results correlated 100%. DOB values showed a strong linear correlation between test systems (Fig. 1A) but was driven primarily by the positive specimens (Fig. 1B and C). All tested specimens in precision studies showed 100% agreement.

### Conclusion

*H. pylori* PyloPlus UBT assay in a pediatric patient population (3–17 years old) was evaluated in this study. The higher prevalence of *H. pylori* infection in male pediatric patients found in our study is consistent with previous reports (7). Our data show that the performance of the PyloPlus UBT assay is equivalent to the BreathTek UBT system and can be used for patients in this age group. The benefit of the PyloPlus UBT system is the lack of a calculation factor needed for resulting as well as the two-way valve system used with the bags which makes collection easier. It is worth noting that the new Meridian Breath ID Urea Breath Test does not include a pediatric calculation adjustment, suggesting that a calculation factor is not required for accurate UBT results in pediatric patients.

Our study had multiple limitations. The breath sample was collected in BreathTek UBT bags and then transferred to PyloPlus UBT bags due to lack of accessibility to the patient by the testing laboratories. However, the DOBs remained comparable after transfer (Fig. 1). The Meridian BreathTek assay required a calculation utilizing age, height, and weight for pediatric patients to calculate UHR for determination of qualitative result, while the PyloPlus does not include any adjustment for pediatric patients. Nevertheless, the qualitative results correlated with comparable DOB values (Fig. 1). Finally, while our patient population spanned the entire age range, it was skewed toward older pediatric patients.
